# A Multi-Satellite Multi-Target Observation Task Planning and Replanning Method Based on DQN

**DOI:** 10.3390/s25061856

**Published:** 2025-03-17

**Authors:** Xiaoyu Xing, Shuyi Wang, Wenjing Liu, Chengrui Liu

**Affiliations:** 1 Beijing Institute of Control Engineering, Beijing 100094, China; xingxiaoyu0904@163.com (X.X.); lwjingbice@163.com (W.L.); liuchengrui_502@163.com (C.L.); 2 National Key Laboratory of Space Intelligent Control, Beijing 100094, China

**Keywords:** satellite observation, task planning, task re-planning, deep reinforcement learning

## Abstract

This paper proposes a task planning method that integrates deep Q-learning network (DQN) with matrix sorting for Earth-oriented static multi-target cooperative observation tasks. The approach addresses emergent satellite failures in imaging constellations by eliminating the need for network model retraining during satellite malfunctions. It enables real-time generation of optimal task allocation schemes in contingency scenarios, ensuring efficient and adaptive task planning. Firstly, a mission scenario model is established by formulating task constraints and defining optimization objectives; secondly, a deep reinforcement learning framework is constructed to output the observation target sequence; then, the observation target sequence is transformed into a target sequence matrix, and a matrix-sorting planning method is proposed to carry out the optimal assignment of the task; lastly, a replanning method is designed for sudden satellite failure and insertion of urgent tasks. The experimental results show that the method has fast task planning speed, high task completion, and immediate task replanning capability.

## 1. Introduction

In recent years, with the rapid development of remote sensing satellite technology and the advancement of constellation networking capabilities, a series of large-scale Earth observation satellite systems have emerged worldwide. For instance, China’s Gaofen satellite constellation, the United States’ Landsat satellite constellation, and commercial constellations such as Jilin-1 and Zhuhai-1 have achieved high-resolution and high-coverage Earth observation capabilities through multi-satellite collaboration. These systems have played a significant role in environmental monitoring, disaster early warning, resource surveying, and other fields. However, with the diversification of observation demands and the surge in the number of targets, it has become increasingly apparent that single-satellite systems have several limitations, such as observation blind spots, long revisit cycles, and conflicts in multi-target observations. There is an urgent need to enhance task completion efficiency and accuracy through multi-satellite collaborative observation mission planning, as well as to promptly revise and replan tasks in the event of satellite failures.

Observation tasks in constellation task planning [1] face the problems of multiple observation targets, large target action spaces, and complex coordination of imaging satellites. With limited resources of imaging satellites, it is a combinatorial optimization problem with multiple constraints and conflicts to achieve the task completion rate within the specified time. Many papers represent the information concerning observation task sequence, execution and observation time in binary or integer encoding, and use meta-heuristic algorithms to solve the problem. Studies [2,3,4,5,6] used genetic algorithms to design unique crossover and mutation strategies to optimize individual tasks, which are able to find the globally optimal solution, but the convergence speed is slow. Studies [7,8,9,10] used particle swarm algorithms to find the optimal solution by iteration from a random solution, which are simple to implement and have fast convergence, but easily fall into local optimal solutions. Studies [11,12,13] used a simulated annealing algorithm, setting the initial solution that meets the task conditions, and iteratively approaching the global optimal solution. This approach has strong local search ability and high solution accuracy, but relies on the selection of the initial solution and has slow convergence speed. However, the aforementioned algorithm is only applicable to predefined scenarios. In the face of changing task information, it requires recalculating and converging to the optimal solution, which is time-consuming, and the algorithm is unable to achieve real-time task replanning

Machine learning algorithms can handle high-dimensional, nonlinear states and action spaces and are suitable for complex dynamic decision-making problems. Zhang et al. [14] built a Markov game framework and used the multi-agent proximal policy optimization algorithm to realize multi-satellite intelligent mission planning. Ou et al. [15] combined deep reinforcement learning (DRL) with a heuristic scheduling method, which can effectively deal with the scheduling problem of satellite shooting ranges. Wei et al. [16] proposed a deep reinforcement learning and parameter transfer-based approach to tackle the multi-objective version of agile earth observation satellite scheduling problems, which reduces the training expenses for its neighboring sub-problems. Liu et al. [17] proposed a Two-stage Multi-Agent deep reinforcement learning-based Distributed computation Offloading (TMADO) framework for a wireless-powered mobile edge-computing network with multiple hybrid access points. It minimized long-term energy provision by jointly optimizing transmit power, wireless power transfer duration, offloading decisions, time allocation, and CPU frequency in a dynamic environment. Feng et al. [18] proposed a multi-satellite cooperative scheduling method for large-scale tasks based on a hybrid graph neural network (GNN) and metaheuristic algorithm, which can achieve an efficient solution to the multi-satellite scheduling problem with tens of thousands of tasks. Jia et al. [19] designed a multi-agent task-offloading and resource allocation algorithm with weighted latency as the optimization goal to adapt dynamic channel variations and fluctuating computational loads on edge-computing satellites. Song et al. [20] proposed a general data-driven framework for solving imaging mission planning problems, and added machine learning algorithms and scheduling algorithms into this framework as core modules. Li et al. [21] presented a multi-agent deep reinforcement learning algorithm with global rewards to solve energy minimization problems for LEO satellite edge-computing networks. However, the current machine learning algorithm takes the observation satellites, task description and other information as the input. If the observation satellites are unable to work in case of sudden failures, the input dimensions of the model will change, which leads to the retraining of the model. It is too time-consuming to satisfy the demands of instantaneous task replanning.

In response to the aforementioned challenges, this paper proposes a method integrating deep Q-learning with matrix-sorting planning (DQNP) for ground-based multi-target collaborative observation tasks. The DQNP framework enables instantaneous mission replanning during unexpected satellite malfunctions, eliminating the need for model retraining to adapt to dynamic contingency scenarios. The main contributions are outlined below:

(1) Intelligent multi-constraint task planning: The model utilizes a DQN to optimize high-dimensional state-action spaces, enabling dynamic prioritization of observation targets in multi-constraint and multi-objective scenarios. The state space encodes real-time task information, and the action space defines observation target selection strategies. A customized reward function integrates task constraints and optimization objectives. Once trained, the model autonomously generates constraint-satisfying observation sequences, significantly enhancing mission completion rates while adhering to complex operational requirements.

(2) Fault-tolerant replanning via modular architecture: The DQNP framework decouples observation target prioritization and satellite-task allocation into two-stage optimization processes. The DQN module generates target sequences based on mission-critical criteria. Subsequently, a matrix-based method assigns available satellites to the prioritized targets. This modular design isolates the selection of imaging satellites from the DQN’s input–output structure, enabling instantaneous mission replanning during satellite failures without model retraining.

The subsequent sections of the paper are structured as follows. In Section 2, we describe the multi-satellite and multi-target observation mission in detail. In Section 3, we describe the model framework of DQNP and the calculation process of each part. In Section 4, we build a simulation mission scenario, simulate satellite failures, and verify the DQNP model by experiments. Section 5 summarizes the research content of the full text and proposes the development direction of future research.

## 2. Multi-Satellite Multi-Target Observation Task Description

### 2.1. Scene Description

This paper focuses on the problem of cooperative observation of static ground-based multi-targets. The imaging satellites employed are agile satellites, denoted as S, which are capable of attitude maneuvers such as pitch, roll, and yaw. These capabilities enable a significantly larger Earth observation range and enhanced Earth observation performance. Limited by orbit parameters and load capacity, a single-satellite system has difficulty meeting the requirements for high spatial–temporal resolution, wide coverage and real-time monitoring of dynamic targets at the same time. Following the approach of reference [22], we assume that each ground target is observed by two imaging satellites. This can enhance the accuracy of three-dimensional information acquisition, expands the coverage area, reduces observation blind spots, and ultimately yields superior observation outcomes. Each observation task is executed only once within the stipulated time frame, eliminating the need for repeated observations.

### 2.2. Description of Parameters and Variables

(1) Model parameters:

***S***—Imaging satellite set, $S=\{S_{1},S_{2},\ldots S_{n}\}$. *n* is the number of imaging satellites.

***T***—Observation target set, $T=\{T_{1},T_{2},\ldots T_{m}\}$. *m* is the number of observation targets.

*TM*—Time frame for implementation of the overall mandate.

*TK*—Set of time windows, $TK=\{t_{1},t_{2},\ldots t_{k}\}$. Discretizing the total *TM* with the principle of invariant visibility of short time regions yields k time windows.

***O***—Visibility matrix. Referring to the literature [22], we obtain the 3D visibility matrix $O\in R^{k\times n\times m}$ of the imaging satellite for the observation target at different moments based on the satellite’s field of view, observation distance, and observation location, where element $O_{t,i,j,}\in [0,1]$ represents the observation effect of satellite *i* on observation target *j* at time *t*, and a larger value indicates a better observation effect.

***Q***—Observation target prioritization set. $Q=\{Q_{1},Q_{2},\ldots Q_{m}\}$

***TN***—The set of time required to observe the target. $TN=\{TN_{1},TN_{2},\ldots TN_{m}\}$

(2) Model variables:

*TS_j_*—Actual observation time for target *j* (s).

*Sf_j_*—The first observation satellite for target *j*.

*Ss_j_*—The second observation satellite for target *j*.

*P_j_*—Task completion marker of target *j*, which takes the values {0,1}.

***H***—Observed target sequence matrix, $H\in R^{m\times 3}$. It contains *m* observation targets, and each row contains {*Sf, Ss, TS*}.

***E***—Observation tasking matrix, $E\in R^{q\times p}$, where *q* is the task execution step, and the number of columns *p* at a task moment includes *n* observation satellites, *n/2* observation targets, and a real time *t_a_*, thus

q=3n/2+1

**Figure 1:**
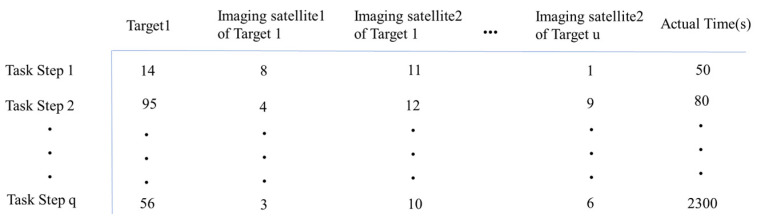
Observation tasking matrix.

Each column actually contains {*T*_1_, *Sf*_1_, *Ss*_1_, *T*_2_, …… *T_n_*_/2_, *Sf_n_*_/2_, *Ss_n_*_/2_, *t*_a_}. Taking Figure 1 as an example, in the matrix shown in the figure, all columns except the last one (time) represent satellite IDs and target IDs. For instance, in task step 1, the observed target is target 14, and the observing satellites are satellite 8 and satellite 11, with an actual observation time of 50 s. This pattern applies similarly to the other task steps.

### 2.3. Optimization Goals

The optimization objectives set for the task of this paper are as follows:

(1) Task planning time:

Time spent on complete task planning *T_p_*.

(2) Task execution time:

Time spent on all observation tasks *T_z_*.

(3) Task completion rate:

$$QT=\frac{\sum_{t=0}^{Tz}V_{t}}{m}$$

*V_t_* refers to the observation task completed at time *t*. *QT* is the number of tasks completed as a percentage of the total number of tasks observed.

(4) Total task reward:

$$R=\sum_{j=1}^{m}R_{j}$$

$R_{j}$ refers to the reward for completing a single observation task, the exact calculation of which is given in the DQN model. *R* refers to the observation effect of all tasks completed.

### 2.4. Constraints

In this paper, we consider four constraints: visibility, time window, on-planet resources, and task conflicts.

(1) Visibility constraint:

To ensure the observation of target *j*, two imaging satellites *i*_1_, *i*_2_ are selected to simultaneously observe a target *j*. Thus, task *j* is observable at moment *t*, which needs to be satisfied:

$$O_{t,i_{1},j}\cdot O_{t,i_{2},j}>0$$

(2) Time window constraint:

Observation tasks for ground-based targets require sustained observations over a period of time and, therefore, need to be performed within the visible time window

$$\left[t_{ja},t_{jb}]\in [t_{js},t_{je}\right]$$

where $t_{ja}$ is the initial observation moment of the target *j*, $t_{jb}$ is the moment of termination of target *j*, $t_{js}$ is the initial visible moment of target *j*, and $t_{je}$ is the terminating visible moment of target *j*.

(3) On-planet resource constraints:

The power and storage capacity of satellite *i* are collectively referred to as on-board resources. The resources consumed to perform observation task *j* include maneuvering energy consumption $E_{m}$ and observation energy consumption $E_{w}$. The available resource of the satellite before the task is $E_{i}$, and it needs to satisfy

$$E_{m}+E_{w}\leq E_{i}$$

(4) Task conflicts:

Due to load limitations, satellites perform only a single observation task at a given time. In order to avoid waste of resources, the same task is executed at most once by two satellites simultaneously. The following conditions need to be satisfied:

$$NW_{t,i}\leq 1$$

$$\sum_{i=1}^{n}NT_{i,j}\leq 2$$

$NW_{t,i}$ is the number of tasks performed by satellite *i* at time *t*, and $NT_{i,j}$ is the number of task executions of satellite *i* for task *j*.

## 3. DQNP Model for Multi-Target Observation Task Planning and Replanning

**Figure 2:**
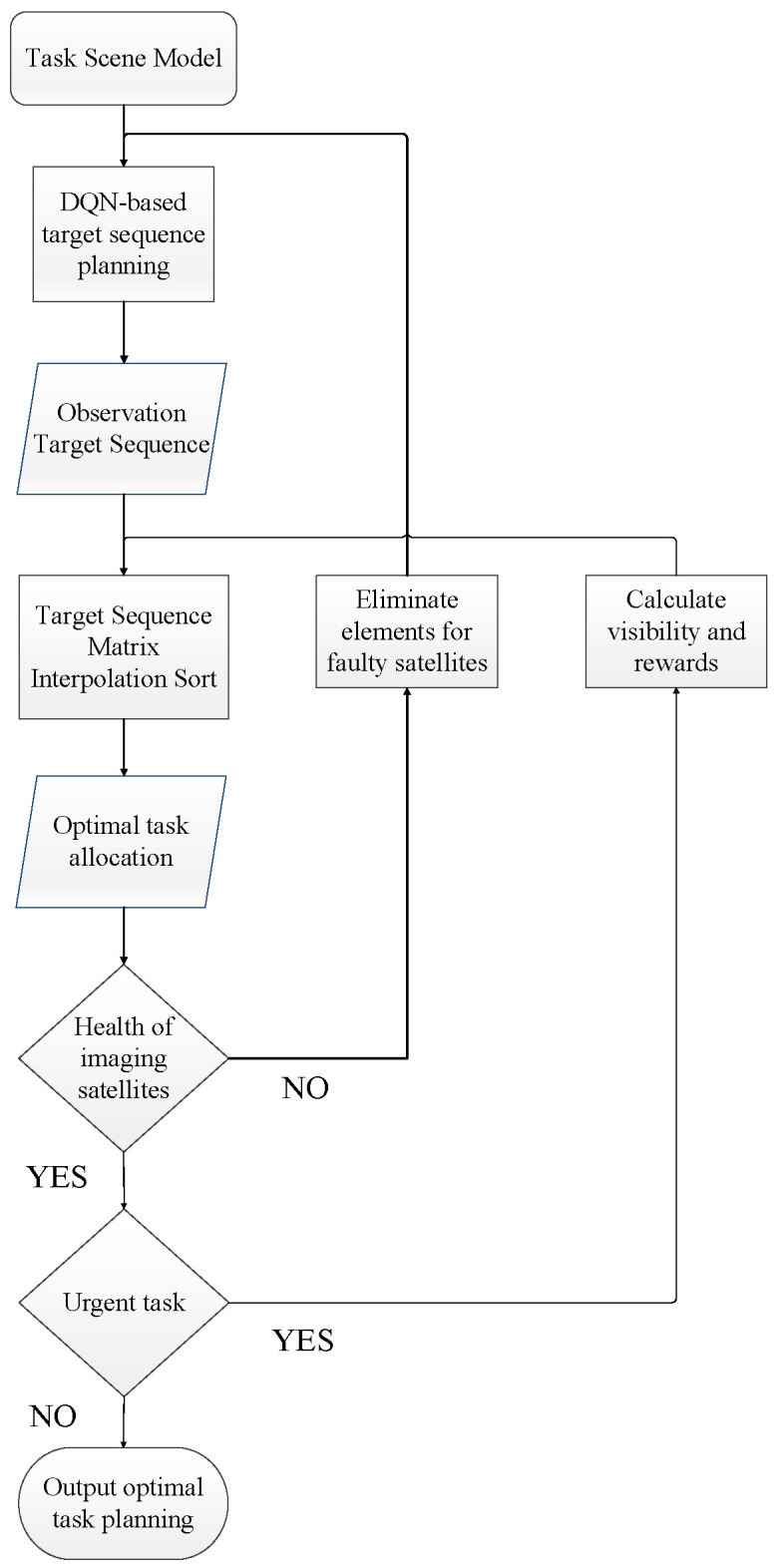
The planning process of DQNP.

The process of DQN-based multi-target observation task planning (DQNP) is as follows: (1) On the basis of constructing the task scenario, the current task description vector is used as the state, and the DQN algorithm is used for optimal action selection to obtain the observation target sequence. (2) The observation target sequence is converted into a target sequence matrix, and the matrix elements are interpolated and stored for conflict resolution and optimal satellite allocation to obtain the final task planning. (3) When an imaging satellite failure is detected, the matrix elements of the failed satellite are eliminated and returned to the first step for task replanning; when an emergency task occurs, the reward and visibility of the emergency task are calculated and inserted into the target sequence matrix for sorting to obtain the task replanning allocation results. The model flow is shown in Figure 2.

### 3.1. DQN-Based Sequence Planning for Observation Targets

**Figure 3:**
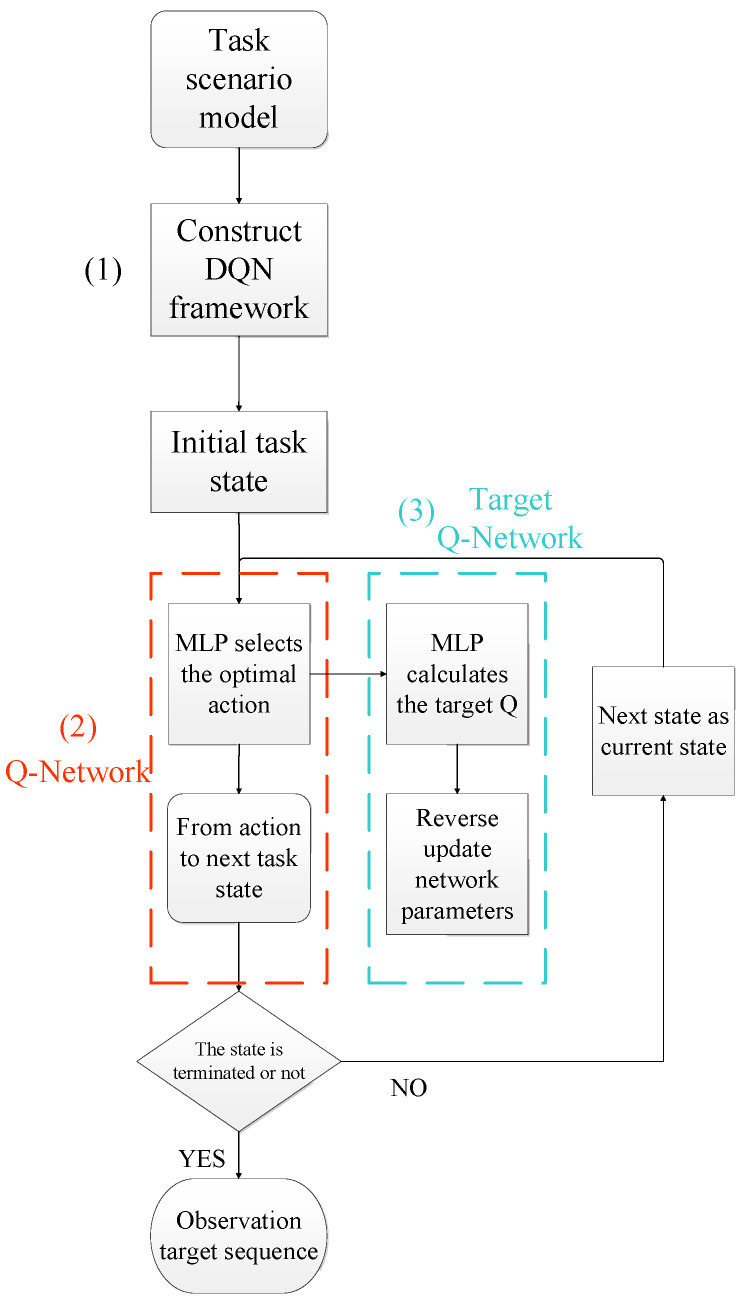
DQN model process.

The preliminary observation target sequence planning using the DQN algorithm is carried out through the following steps: (1) A deep reinforcement learning framework for multi-target observation task planning is constructed. Based on the constructed task scenario, the environment, state, action, and reward of the DQN algorithm are configured according to the specific task constraints and requirements. (2) The optimal actions are predicted and selected using the Q-value network. A Multilayer Perceptron (MLP) is chosen as the Q-value network, where the task description vector of the current state is input into the Q-value network. The MLP aggregates the task information and outputs the optimal action prediction vector. The action vector leads to the next state, ultimately outputting the observation target sequence. (3) The value function is fitted using the target Q-network. A MLP model is selected as the target Q-network to calculate the target Q-value based on the state information of the next task. The output action value is used to update the neural network parameters through backpropagation. The preliminary observation target sequence planning process based on DQN is illustrated in Figure 3.

#### 3.1.1. Framework Construction of the DQN Model

The DQN algorithm is used for initial observation target sequence planning, and the framework of deep reinforcement learning algorithm is built first. The basic elements of the framework are set according to the content of the task scene, including environment, state, action, and reward.

**Environment.** The task scene is used as an environment to interact with the intelligences, and the states and actions are provided according to the actual task requirements and imaging satellite information. Based on the completion of the scene modeling, the visibility matrix ***O*** is used as the environment matrix for linking states and actions.

**State.** The total task completion is taken as the state, and the most critical task description variables are selected as the features of the state vector. There are *m* observation targets in total, and each observation target *j* mainly contains six variables {*Q_j_*, *TN_j_*, *TS_j_*, *Sf_j_*, *Ss_j_*, *P_j_*}, which are arranged to obtain the matrix $U\in R^{m\times 6}$. Due to the different units and magnitudes of different variables, in order to ensure the training effect of the subsequent neural network, ***U*** should be normalized and flattened. The state vector $X\in R^{6m+1}$ is obtained by normalizing ***U***, and the current time *t* is added as the last column. The formula is as follows:

$$X_{6i+j}=\frac{U_{i,j}-\min_{k\in [0,m]}(U_{i,k})}{\max_{k\in [0,m]}(U_{i,k})-\min_{k\in [0,m]}(U_{i,k})}$$

**Action.** We set the execution of a task as an action and set the action vector $A\in R^{m}$, where $A_{i}\in (0,1)$. We then select the serial number corresponding to the maximum value as the mission target to be observed at the next moment:

$$a=\arg\max_{i\in [0,m]}(A_{i})$$

**Reward.** Reward is the basis for judging the advantages and disadvantages of selecting action *a* in the current state *X*. It needs to be set artificially according to the current task state, task constraints and optimization goals. Positive rewards are set when a task with high priority, good observation effect, less resource consumption and first execution is performed; negative rewards are set when the same task is performed repeatedly. The formula is as follows

$$R=\left\{\begin{array}{cc} (O_{t,a,S_{fa}}+O_{t,a,S_{sa}}+Q_{a}-0.1TS_{a})/3, & P_{a}\neq 1\\ -1, & P_{a}=1 \end{array}\right.$$

We convert the variables in the formula to the elements in the state vector ***X*** as follows:

$$R=\left\{\begin{array}{c} (O[t][a][X_{6a+3}]+O[t][a][X_{6a+4}]+X_{6a}-0.1X_{6a+2})/3,\;X_{6a+5}\neq 1\\ -1,\;\;\;\;X_{6a+5}=1 \end{array}\right.$$

#### 3.1.2. Q-Network-Based Action Selection

We first initialize the task state ***X*_0_** as the current state and compute the reward *R*. We then select the MLP as the Q-network, initialize the Q-network parameter *w*, set the experience playback pool ***D*** and empty it.

The current state ***X*** is used as the input vector of the Q-network. Each element of ***X*** is multiplied with the weights of the first implicit layer, respectively, and then summed. The bias value *b*_1_ is added after going through the activation function to obtain the output ***h*_1_** of the layer. The Softmax function is chosen as the activation function *f*_1_, which is expressed as follows:

$$h_{1}=f_{1}(\theta_{1}X+b_{1})$$

We use the output of implicit layer *1* as the input of the next implicit layer *2*, repeat the above equation, and finally output an action corresponding to the action value *Q*. We then use ε-greedy algorithm to select the corresponding action *a* in the current *Q* value output. The action *a* is executed in the state ***X*** to obtain the next state ***X′*** and its reward *R*′. We then place the quintuple {***X***, *a*, *R*, ***X′***, *R*′} into the experience playback pool ***D***.

We determine whether the next state ***X′*** is the termination state: if yes, we stop the current round of iteration, and output the target sequence of this observation; otherwise, we take ***X′*** as the current state ***X***, and repeat the above steps.

#### 3.1.3. Parameter Update Based on Target Q-Network

Whenever the number of samples in the experience playback pool meets the set value, *p* small batches of samples are randomly selected for parameter updating of the neural network. Firstly, the target Q network is set up to calculate the target *Q* value, with the same structure as the Q network and the parameter *w’*. We input the next state ***X′*** and obtain the action *Q* value *Q*(***X′***, *a′*; *w′*) of the next state, and the target *Q* value is as follows:

$$Q_{t\arg ert}=R+\gamma \max_{a^{\prime }}Q(X^{\prime },a^{\prime },w^{\prime })$$

γ is the discount factor, which takes the value in the range of (0,1). The loss function uses Mean Square Error (MSE).

$$MSE=\frac{1}{p}\sum_{j=1}^{p}\left(Q_{t\arg et}-Q(X^{\prime },a^{\prime },w^{\prime })\right)$$

All parameters *w* of the Q network are updated by back propagation of the gradient of the neural network every *m* iterations, and the parameters of the target Q-network are updated by replicating *w*. This design provides a relatively stable target value and helps to reduce instability during training.

### 3.2. Optimal Task Planning Using Matrix Ordering

**Figure 4:**
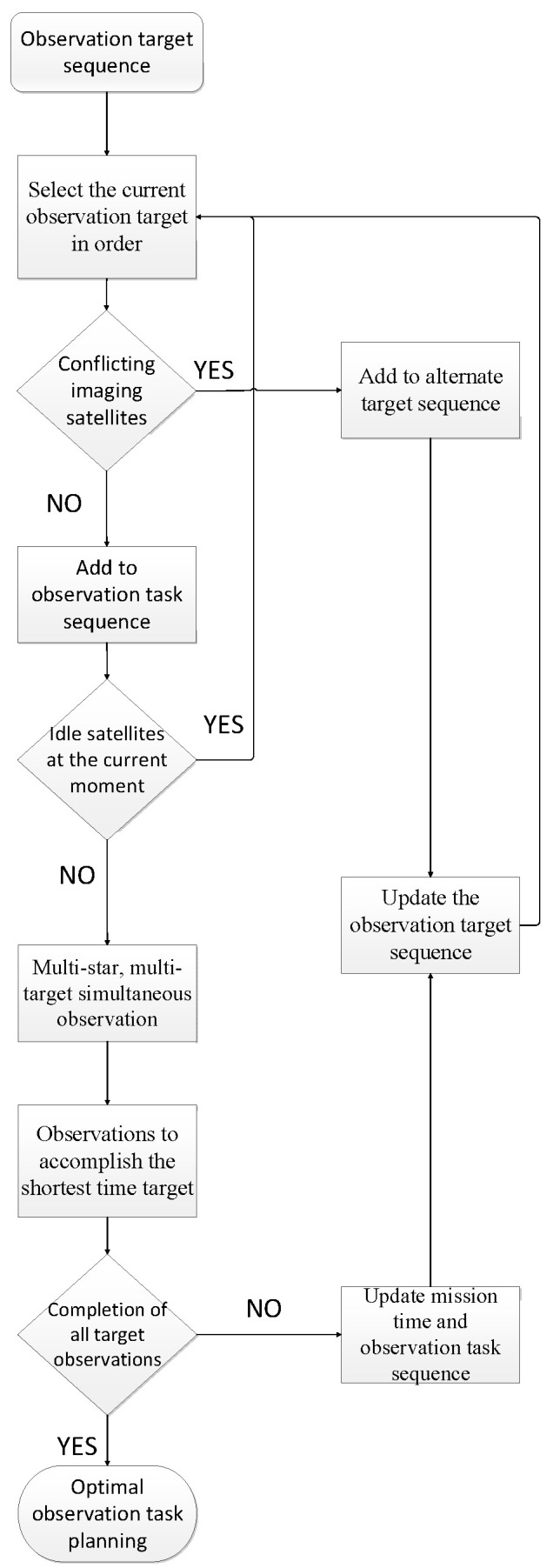
Optimal task planning process.

In this paper, we use a matrix to store the relevant task information, and compare the matrix elements to facilitate the sorting and storage. Firstly, the observed target sequence output in Section 2.1 is converted into a target sequence matrix $H\in R^{m\times 3}$. We set up the all-zero alternate target sequence $B\in R^{v\times 3}$ with the same structure as ***H***. We then set up the observation task execution sequence matrix $E\in R^{q\times p}$. The steps of optimal task planning using matrix ordering are as follows: (1) We select the observation target *j* information *H_j_* in ***H*** in order, and compare its imaging satellites with the elements in *E_k_* corresponding to the current step *k*. If there is no conflict, we add *H_j_* to *E_k_*; otherwise, we add *H_j_* to ***B***. (2) We determine whether there is a vacant satellite in *E_k_*. If not, the task is executed; otherwise, we jump back to step 1. (3) We select the target with the shortest observing time in *E_k_*, and delete it after completion. If all tasks complete the observation, the final task planning is output. Otherwise, the unfinished task is left to the next task moment *E_k_*_+1_, the time information is updated with the observation task sequence, and Steps 1 and 2 are repeated. The flow of the algorithm is shown in Figure 4, and the pseudo-code is shown in Table 1.

### 3.3. Task Replanning for Contingencies

(1) Satellite failure conditions:

When *k* imaging satellites fail, the following points in the task planning step need to be modified and then the task planning process repeated:

① Take the set of faulty satellites as ***F*** = {faulty satellite 1, faulty satellite 2, …… faulty satellite *k*}, and the set of healthy satellites as $S^{\prime }=\{S_{1},S_{2},\ldots S_{n-k}\}$. Set the corresponding element of the visibility matrix of the faulty satellites to zero,

$$O_{t,i}=0,\;i\in F$$

② For the observed target sequences output from the DQN model, the sequence elements *H_i,j_* containing the faulty satellites need to be modified according to ***O***. The selection is based on the following:

$$H_{i,j}=\max_{b\in S^{\prime }}(O_{t,b,i})$$

③ The number of observation targets *lm* that can be executed simultaneously in optimal task planning becomes

$$lm=\left\lfloor \frac{n-k}{2}\right\rfloor$$

$\left\lfloor \cdot \right\rfloor$ denotes downward rounding. The number of columns *q* of the final task sequence ***E*** is changed to

q=3*lm+1

(2) Urgent task planning:

When an urgent task *k* occurs, its visibility needs to be calculated then the task reward is calculated according to the following equation,

$$R_{k}=(O[t][Sf_{k}][k]+O[t][Ss_{k}][k]+P_{k}-0.1TN_{k})/3$$

It is compared with the current observation target reward value and inserted into the observation target sequence ***H*** in order. Then we recalculate ***E*** based on the updated ***H***.

## 4. Experimental Results and Discussion

### 4.1. Scene Construction

**Figure 5:**
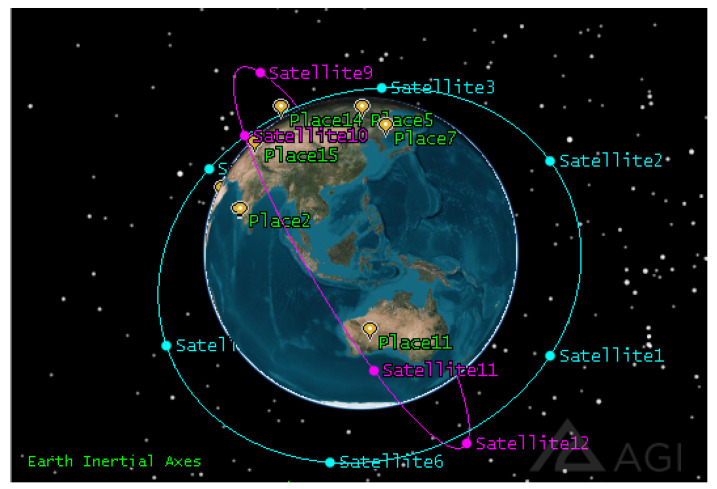
Three-dimensional scene.

**Figure 6:**
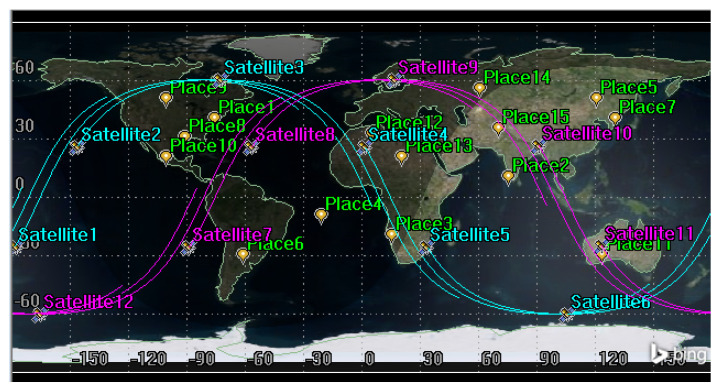
Tracks of subsatellite points.

We set up two satellite orbits with six satellites operating at a phase difference of 60 degrees in each orbit, totaling *n* = 12 imaging satellites. We randomly select *m* = 100 ground observation targets at 60° S–60° N, and the task time limit is 1200 s. In order to better display the effect, we selected 15 of the 100 observation targets for display. The orbit data are shown in Table 2, the 3D scene is shown in Figure 5, and the tracks of subsatellite points are shown in Figure 6.

### 4.2. Model Training

**Figure 7:**
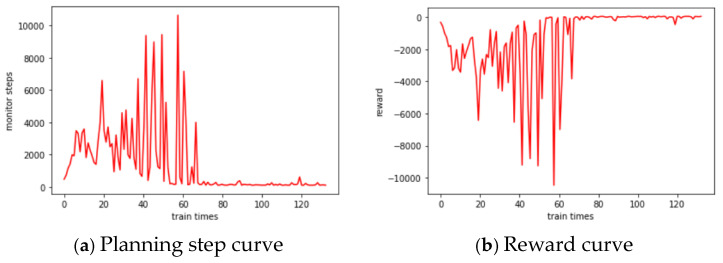
DQN training process curves.

The computer configuration CPU used in this experiment is the i5 processor and the GPU is the GTX1060. First, we train the DQN model, whose input state vector of the MLP network is 701 dimensions, while the output predictive action vector is 12 dimensions. The number of neurons contained in the three implicit layers is set to 1024, 512, and 512, and the learning rate *lr* = 0.001. The number of training times is set to 40,000, with a total of 100 observation targets. Therefore, when the number of action steps of each training is 100, it is regarded as an optimal planning, and when the number of optimal plannings reaches 20, it is regarded as the completion of training. The actual training reaches the termination condition 131 times, which takes 2336 s, and the total reward is 63.27. The observed action curve of the intelligent body is shown in Figure 7a, and the reward learning curve is shown in Figure 7b:

### 4.3. Task Planning Results

**Figure 8:**
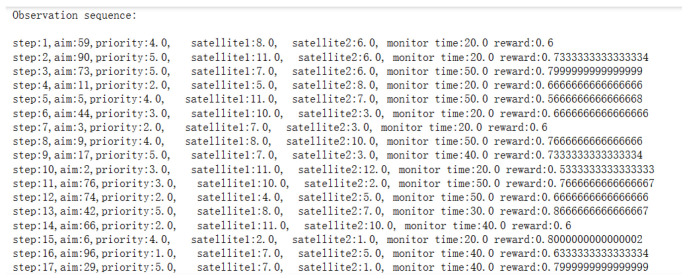
Part of observation target sequence.

**Figure 9:**
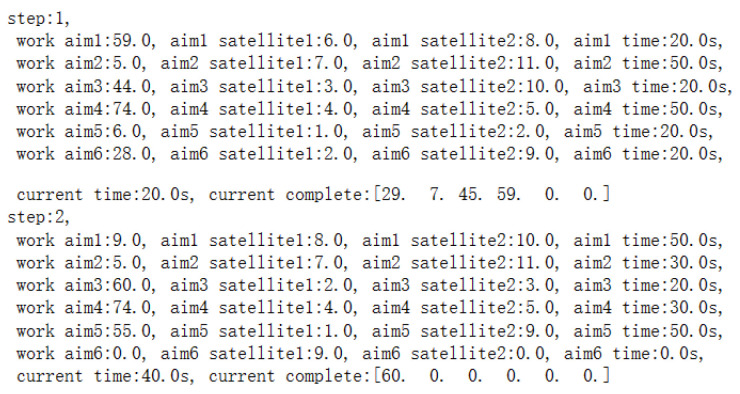
Part of the optimal task planning sequence.

**Figure 10:**
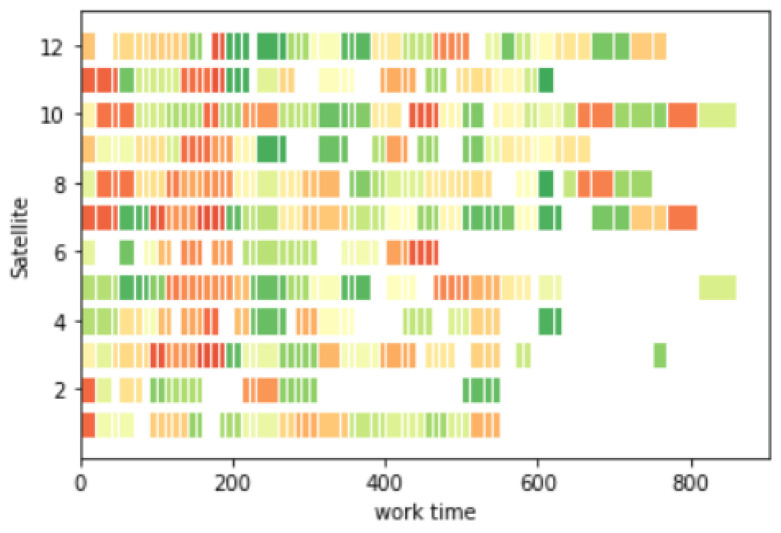
Gantt chart for task planning.

Firstly, the trained DQN model is utilized to obtain the observation target sequence, with part of the process shown in Figure 8:

According to the observation target sequence matrix, the optimal task planning is carried out, and part of the process is shown in Figure 9:

Task planning took 0.57 s; task execution took 890 s; and the task planning Gantt chart is shown in Figure 10. Different colors in the picture represent different observation targets.

Since the current intelligent planning algorithms do not have instant replanning capability, it is not possible to compare the present method with them. In order to verify the optimization effect of the method adopted in this paper on the task objectives, the traditional completion speed priority algorithm and the observation effect priority algorithm are selected to compare the effect. The speed priority algorithm involves prioritizing spare satellite tasks, which may ignore the observation effect; the effect priority algorithm involves prioritizing satellite tasks with good observation effect, which may lead to task conflict and prolong the execution time. The results are shown in Table 3:

According to the above table, it can be seen that the speed priority algorithm has a short task execution time and a fast observation speed, but it cannot guarantee the observation effect; the effect priority algorithm has a high task reward, but a poor task completion rate and a long task execution time. Compared with the above two methods, the DQNP proposed in this paper has higher task reward and shorter task execution time, which is in line with the optimization objective.

### 4.4. Results of Task Replanning

(1) Satellite failure scenarios

**Figure 11:**
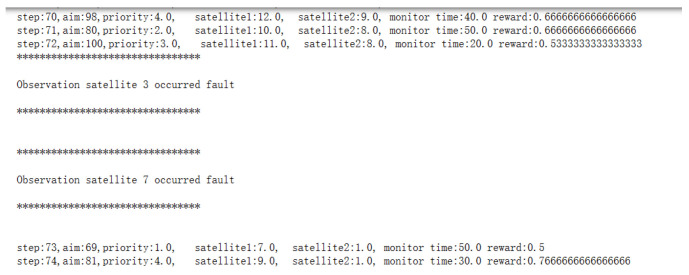
Part of the satellite failure replanning process.

**Figure 12:**
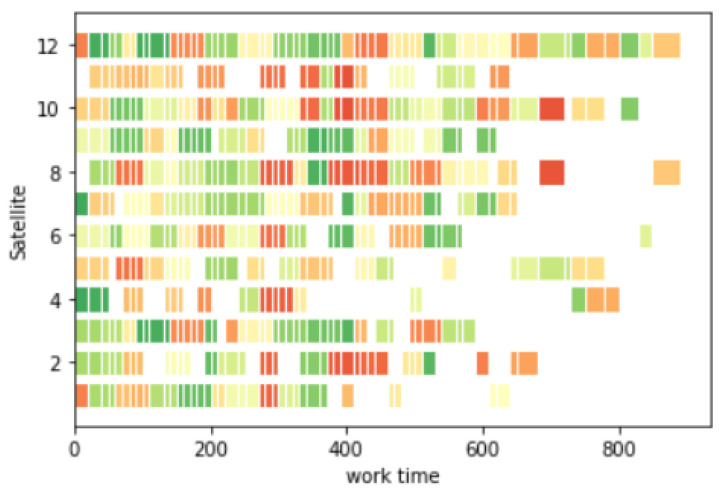
Gantt chart for satellite fault replanning.

Assuming that the imaging satellite 3 and imaging satellite 7 fail to execute the task at 600 s of task execution, task replanning is performed for them. The replanning time is 0.58 s; the task execution time is 930 s; and the task reward is 61.56. Part of the process is shown in Figure 11. The Gantt chart is shown in Figure 12:

(2) Emergency task

**Figure 13:**
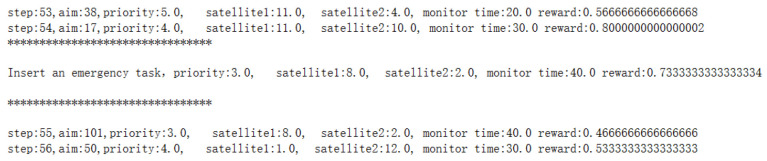
Part of the urgent task replanning process.

**Figure 14:**
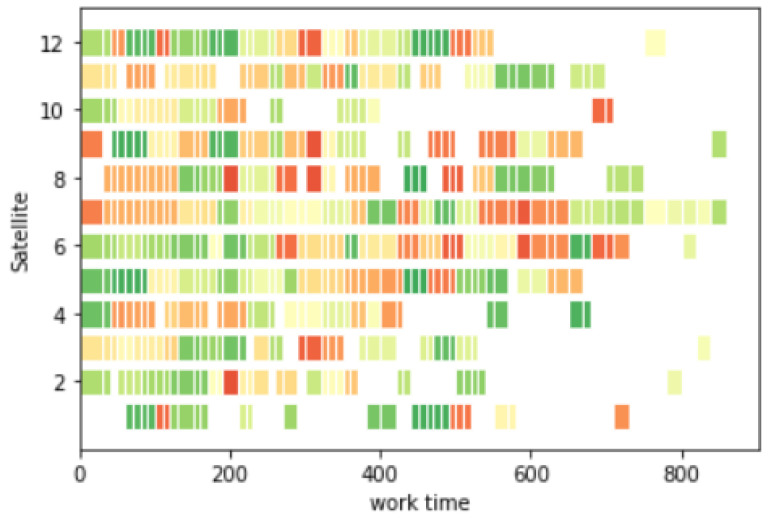
Gantt chart for urgent task replanning.

Suppose an urgent task is inserted at 500 s and task replanning is performed for it. The urgent task is numbered 101; the priority is 3; the final replanning time is 0.61 s; the task execution time is 950 s; and the task reward is 62.42. Part of the process is shown in Figure 13, and the Gantt chart is shown in Figure 14.

A comparison of task completion for the three scenarios is shown in Table 4.

As can be seen from the comparisons in the table above, in the case of the failure of imaging satellites 3 and 7, fewer imaging satellites become available, resulting in longer task execution times and reduced observation effectiveness. Therefore, the total task reward is reduced. In the case of inserting an urgent task, the current task execution has to be interrupted for task replanning, which leads to a decrease in the observation effect, an increase in the task planning time and a decrease in the total task reward. Moreover, due to the addition of observation tasks, the task execution time increases accordingly. According to the above, the replanning method proposed in this paper is able to complete the task planning and execution within the stipulated time, even in the case of satellite failure and insertion of an emergency task, and has a high task completion rate and completion effect.

## 5. Conclusions

In this paper, we propose a multi-satellite multi-target observation task planning model, DQNP. It possesses the ability of intelligent multi-constraint task planning via DQN and fault-tolerant replanning via matrix-sorting planning. Based on experimental verification, this method has demonstrated fast task planning speed and high task completion, and possesses the ability of instant task replanning. This approach ensures real-time task reallocation and scheduling optimization, thereby sustaining operational continuity and enhancing system robustness against equipment anomalies under time-critical constraints.

However, a limitation of the DQN-based approach (DQNP) is that, as the number of observation targets increases, the dimensions of the action and state spaces expand, leading to exponential growth in computational complexity. This results in time-consuming training and challenges in achieving task allocation for a larger number of observation targets. In future work, we aim to address this issue by reducing vector dimensions through dimensionality reduction or feature extraction techniques, thereby enabling large-scale observation task allocation for ground targets.

## Figures and Tables

**Table 1:** Optimal task planning algorithm using matrix sorting.

Convert the observed target sequence into a target sequence matrix ***H*** Initialize the task sequence matrix ***E*** = {} Initialize the alternate target sequence matrix ***B*** = {} While ***H*** ≠ 0 do For i = 1, 2, … q While ***E****_i_* has zero elements do If ***H*_1_** has no task conflicts with stored targets in ***E****_i_* Store ***H*_1_** to the vacant position of ***E_i_*** Else Store ***H*_1_** to ***B*** Delete ***H*_1_** from ***H*** End while Complete the task with the shortest time *E_i_*_+1_ = *E_i_*, delete completed task element and update task time Add ***B*** to ***H*** in top order End for End while

**Table 2:** Orbital data.

Orbit	Semimajor Axis (km)	Eccentricity	Inclination (deg)	Argument of Perigee (deg)	RAAN (deg)
1	8878.14	0.00081	60.0144	270.11	0.24357
2	8878.14	0.00082	60.1367	269.698	89.9366

**Table 3:** Comparison of experimental effects of models.

Model	Task Planning Time (s)	Task Execution Time (s)	Task Completion Rate	Total Task Rewards
DQNP	0.57	890	100%	65.26
Speed priority	0.36	740	100%	41.68
Effect priority	0.42	1460	89%	52.96

**Table 4:** Results of task planning in different situations.

Situation	Task Planning Time (s)	Task Execution Time (s)	Task Completion Rate	Total Task Rewards
Normal	0.57	890	100%	65.26
Satellite failure	0.58	930	100%	57.56
Urgent task	0.61	950	100%	64.42

## Data Availability

Due to the identity of the organization, the procedures and data need to be kept confidential.

## References

[1] Kennewell J.A., Vo B.N. An overview of space situational awareness. Proceedings of the 16th International Conference on Information Fusion.

[2] Chen Y., Xu M., Shen X., Zhang G., Lu Z., Xu J. (2020). A multi-objective modeling method of multi-satellite imaging task planning for large regional Mapping. Remote Sens..

[3] Fei H., Zhang X., Long J., Liu L., Wang Y. (2023). Towards Multi-Satellite Collaborative Computing via Task Scheduling Based on Genetic Algorithm. Aerospace.

[4] Gan L., Gong S. (2021). Observation mission planning for maneuverable satellite constellations towards multiple targets. J. Tsinghua Univ. (Sci. Technol.).

[5] Xu Z., Liu Y., Feng Z. (2022). Mission Planning for Astronomical Satellite Based on Genetic Algorithm under Tiling Coverage Strategy. Chin. J. Space Sci..

[6] Ding Y.N., Lei Y.J., Wang S.Y., Huang P.X. (2024). Agileimagingsatellitetaskplanningmethodforrevisittimerequirements. Aerosp. Control Appl..

[7] Lu Z., Shen X., Li D., Li D., Chen Y., Wang D., Shen S. (2023). Multiple super-agile satellite collaborative mission planning for area target imaging. Int. J. Appl. Earth Obs. Geoinformation.

[8] Chen Y., Tian G., Guo J., Huang J. (2021). Task Planning for Multiple-Satellite Space-Situational-Awareness Systems. Aerospace.

[9] Liu L.H., Dong Z.H., Su H.X., Chen G.Z. (2023). Multi-satellite distributed mission scheduling via game strategy. Control Theory Appl..

[10] Chen H., Li L., Zhong Z., Li J. (2015). Approach for earth observation satellite real-time and playback data transmission scheduling. J. Syst. Eng. Electron..

[11] Stollenwerk T., Michaud V., Lobe E., Picard M., Basermann A., Botter T. (2021). Agile Earth Observation Satellite Scheduling With a Quantum Annealer. IEEE Trans. Aerosp. Electron. Syst..

[12] Long J., Wu S., Han X., Wang Y., Liu L. (2023). Autonomous Task Planning Method for Multi-Satellite System Based on a Hybrid Genetic Algorithm. Aerospace.

[13] Zhou Q.R., Wan G.H. (2022). Modeling and optimization algorithm of multi-task assignment for multi-satellite. Aerosp. Control Appl..

[14] Zhang G., Li X., Hu G., Li Y., Wang X., Zhang Z. (2023). MARL-Based Multi-Satellite Intelligent Task Planning Method. IEEE Access.

[15] Ou J., Xing L., Yao F., Li M., Lv J., He Y., Song Y., Wu J., Zhang G. (2023). Deep reinforcement learning method for satellite range scheduling problem. Swarm Evol. Comput..

[16] Wei L., Chen Y., Chen M., Chen Y. (2021). Deep reinforcement learning and parameter transfer based approach for the multi-objective agile earth observation satellite scheduling problem. Appl. Soft Comput..

[17] Liu X., Chen A., Zheng K., Chi K., Yang B., Taleb T. (2024). Distributed Computation Offloading for Energy Provision Minimization in WP-MEC Networks with Multiple HAPs. IEEE Trans. Mob. Comput..

[18] Feng X., Li Y., Xu M. (2024). Multi-satellite cooperative scheduling method for large-scale tasks based on hybrid graph neural network and metaheuristic algorithm. Adv. Eng. Inform..

[19] Jia M., Zhang L., Wu J., Guo Q., Zhang G., Gu X. (2024). Deep Multi-Agent Reinforcement Learning for Task Offloading and Resource Allocation in Satellite Edge Computing. IEEE Internet Things J..

[20] Song Y.-J., Zhou Z.-Y., Zhang Z.-S., Yao F., Chen Y.-W. (2020). A framework involving MEC: Imaging satellites mission planning. Neural Comput. Appl..

[21] Li H., Yu J., Cao L., Zhang Q., Song Z., Hou S. (2024). Multi-agent reinforcement learning based computation offloading and resource allocation for LEO Satellite edge computing networks. Comput. Commun..

[22] Kocaman S.A. (2008). Sensor Modeling and Validation for Linear Array Aerial and Satellite Imagery. Ph.D. Thesis.

